# Secondhand smoke increases the risk of developing chronic obstructive pulmonary disease

**DOI:** 10.1038/s41598-024-58038-2

**Published:** 2024-03-29

**Authors:** Wen-Chi Su, Huai-Lei Juan, Jia-In Lee, Shu-Pin Huang, Szu-Chia Chen, Jiun-Hung Geng

**Affiliations:** 1 Department of Internal Medicine, Kaohsiung Medical University Hospital, Kaohsiung Medical University, Kaohsiung, Taiwan; 2 Department of Internal Medicine, Kaohsiung Municipal Siaogang Hospital, Kaohsiung Medical University, Kaohsiung, Taiwan; 3 Division of Pulmonary and Critical Care Medicine, Department of Internal Medicine, Kaohsiung Medical University Hospital, Kaohsiung Medical University, Kaohsiung, Taiwan; 4 Department of Psychiatry, Kaohsiung Medical University Hospital, Kaohsiung Medical University, Kaohsiung, Taiwan; 5 Graduate Institute of Clinical Medicine, College of Medicine, Kaohsiung Medical University, Kaohsiung, Taiwan; 6 Department of Urology, Kaohsiung Medical University Hospital, Kaohsiung Medical University, Kaohsiung, Taiwan; 7 Ph.D. Program in Environmental and Occupational Medicine, College of Medicine, Kaohsiung Medical University, Kaohsiung, Taiwan; 8 Institute of Medical Science and Technology, College of Medicine, National Sun Yat-Sen University, Kaohsiung, Taiwan; 9 Division of Nephrology, Department of Internal Medicine, Kaohsiung Medical University Hospital, Kaohsiung Medical University, Kaohsiung, Taiwan; 10 Faculty of Medicine, College of Medicine, Kaohsiung Medical University, Kaohsiung, Taiwan; 11 Research Center for Environmental Medicine, Kaohsiung Medical University, Kaohsiung, Taiwan; 12 Department of Urology, Kaohsiung Municipal Siaogang Hospital, No. 482, Shanming Rd, Xiaogang District, Kaohsiung City, 812 Taiwan

**Keywords:** Environmental impact, Respiratory tract diseases

## Abstract

Smoking is the most important risk factor for chronic obstructive pulmonary disease (COPD), however evidence from large-scale studies on whether secondhand smoke (SHS) increases the risk of COPD is still lacking. We conducted this large longitudinal study to investigate the association between SHS and the development of COPD. This is a longitudinal study. Data on 6519 subjects who were never-smokers, had no history of COPD, and had complete lung function records were extracted from the Taiwan Biobank. They were divided into two groups according to SHS exposure: no exposure and exposure groups. Data were collected when participants enrolled in the study and during regular follow-up. Cox proportional hazards regression models were used to estimate the relative risk (RR) and 95% confidence interval (CI) for the association between SHS and the risk of developing COPD. At 48 months of follow-up, 260 (4%) participants in the no exposure group and 34 (7%) participants in the exposure group developed COPD. The RR of incident COPD development was significantly higher in the exposure group than that in the no exposure group after adjusting for confounders (RR = 1.49; 95% CI 1.04 to 2.14; P value = 0.031). There is a dose–response relationship between the duration of exposure to SHS and the risk of incident COPD, which demonstrates that an additional hour of exposure to SHS per week was associated with a 1.03-fold increased likelihood of developing COPD after adjusting for confounders (RR = 1.03; 95% CI 1.00 to 1.05; P value = 0.027). SHS exposure contributes to the development of COPD. This finding can help raise awareness of the harms of SHS and provide a reference for formulating anti-smoking policies.

## Introduction

Chronic obstructive pulmonary disease (COPD) is a major cause of morbidity and mortality worldwide and is expected to become the 4th leading cause of disability-adjusted life years (DALY) by 2030^1,2^. Although the reported prevalence of COPD varies between countries due to differences in definitions and methodologies, the global prevalence in adults aged over 30 was estimated to be 10.3% in 2019, which translates to 391.9 million people^3^. The Asia–Pacific region bears the highest burden of COPD in terms of deaths, DALY, and years of life lost (YLL)^1^. In Taiwan, the reported prevalence of COPD in adults aged over 40 years ranges from 2.48% to 9.5%^4^. According to 2021 statistics published by the Ministry of Health and Welfare in Taiwan, chronic lower respiratory tract diseases are the 8th leading cause of death, associated with an estimated mortality rate of 26.6 per 100,000 people. The same source indicated that COPD morbidity and mortality resulted in a total of 9.5 YLL in individuals below the age of 70 years, making it the 10th leading cause of YLL in 2017. The prevalence of COPD is high and is expected to keep growing due to cumulative exposure to risk factors and population aging, leading to increased psychological, economic, and social burden in the coming decades^1,5^.

Tobacco smoke remains the most significant cause of COPD, however an estimated 25–45% of patients with COPD have never smoked^6^. Other risk factors associated with the disease include genetics, childhood respiratory infections, asthma, and various environmental exposures such as occupational dust, outdoor and indoor air pollutants, and biomass fuels^7^. Second-hand smoke (SHS), which is another form of biomass smoke, has been linked to coronary heart disease, stroke, and lung cancer in adult non-smokers^8^. Recent studies have also linked SHS to a higher incidence and prevalence of chronic kidney disease and an increased overall risk of cancer, particularly lung and breast cancer, in never-smokers^9,10^.

According to an analysis of data from the 2019 Global Burden of Diseases (GBD) report, approximately 1.3 million fatalities worldwide were attributed to SHS exposure in 2019^11^. The morbidity impact associated with SHS exposure, assessed through DALYs, revealed regional disparities, with elevated rates observed in low-income countries across Southeast Asia and the eastern Mediterranean region compared to Europe^11^. Moreover, an estimated 40% of children (aged 0–14 years), 33% of non-smoking men, and 35% of non-smoking women worldwide were reported to be exposed to SHS across various environments on a global scale^12^. Among adults, asthma and ischemic heart disease were identified as the predominant health concerns, while lower respiratory infections were the primary concern among children^13^. In addition, a study published in Nature Medicine in 2024 demonstrated that exposure to SHS increased the likelihood of ischemic heart disease, stroke, type 2 diabetes, and lung cancer by approximately 8%, 5%, 1%, and 1%, respectively^14^. Furthermore, evidence suggests detrimental associations between SHS and otitis media, asthma, lower respiratory infections, breast cancer and COPD^14^. These studies underscore the global imperative for individuals, governments, and the World Health Organization to recognize the urgency of controlling SHS.

Although the prevalence of SHS exposure has decreased substantially in Taiwan in the past decades^15^, SHS still poses a significant socioeconomic burden. A study evaluating the cost of smoking and SHS in Taiwan found that direct costs for treating tobacco-related diseases amounted to US$828 million in 2010, accounting for 3.4% of total personal healthcare expenditure. Furthermore, adding indirect costs measured by the value of lost productivity resulted in a total of US$1.67 billion, representing 0.4% of the gross domestic product in Taiwan^15^.

Previous studies have explored the relationship between SHS and COPD, and shown that SHS exposure is related to higher mortality from COPD^16,17^. In addition, in COPD patients who are not currently smoking, SHS exposure has been shown to cause higher exacerbation rates, worse respiratory symptoms, and poorer health status^18–20^. Both the Singapore Chinese Health Study and a 17-year cohort study in China corroborate these findings, demonstrating the adverse impact of SHS exposure on COPD risk^16,21^. However, conflicting evidence exists, with certain studies reporting no significant associations between SHS and COPD^22–26^. Notably, a case–control study in the United States^22^ and research involving COPD patients in Hong Kong^23^ found no increased risk associated with SHS exposure. Despite these discrepancies, the potential for SHS to cause COPD in non-smokers remains a subject of debate. Given the limited scope of current evidence, particularly from small cohorts, cross-sectional studies, and Western countries, there is a critical need for large-scale longitudinal studies in the Asia–Pacific region to comprehensively investigate the relationship between SHS and COPD. Therefore, the aim of this large-scale longitudinal study was to investigate the association between SHS and the development of COPD in Taiwan.

## Methods

### Data source and study population

**Figure 1:**
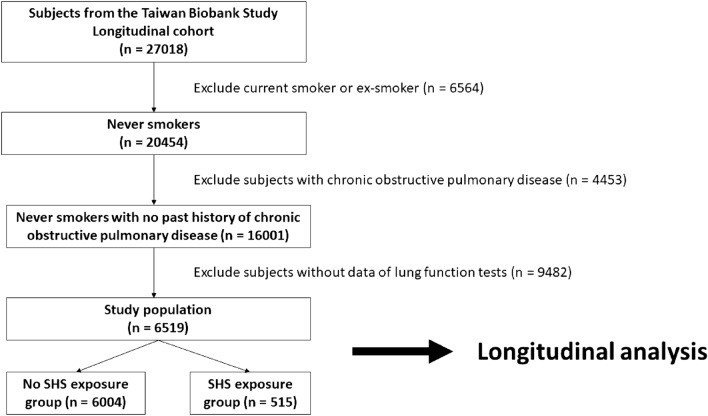
Selection of study population.

This study utilized the Taiwan Biobank (TWB), a substantial community research database, which has recruited cancer-free volunteers aged 20 to 70 years from more than 30 recruitment centers in Taiwan since 2008. Upon enrollment, individuals underwent questionnaire surveys, physical examinations, blood sampling, and lung function tests. Starting from 2016, recurrent assessments of these characteristics have been conducted during scheduled visits, typically occurring within 2–4 years following the initial appointment. By August 2021, the biobank had amassed a cumulative count of 151,406 participants, with 37,508 having undergone the initial follow-up assessment^27^. Detailed information about its methodology and development has been reported in previous studies^28–32^. As a result of the TWB releasing data in batches, with varying release dates and prices, we encountered difficulties in obtaining data for all subjects who completed the initial follow-up assessment. Consequently, a total of 27,018 follow-up participants were enrolled in the present study (Fig. 1). After excluding active and ex-smokers, individuals with a history of COPD, and those without complete lung function test results, the analysis included 6,519 participants (Fig. 1). Written informed consent was obtained from all participants, and the study adhered to the principles of the Declaration of Helsinki. The Institutional Review Board of Kaohsiung Medical University Hospital approved this study (Approval No. KMUHIRB-E(I)-20190398).

The participants were first classified into three groups based on their smoking status, namely "never-smokers," "ex-smokers," and "active smokers," using self-reported questionnaires. The never-smokers were also asked about their exposure to SHS: "Have you been exposed to SHS?" Those who reported exposure to SHS were assigned to the exposure group, while the others were assigned to the no exposure group. Subsequently, participants in the exposure group were asked additional questions regarding their SHS exposure, including "How many hours per week have you been exposed to SHS?" and "Is your exposure to SHS at home or work or both?".

### Variables and study outcomes

We gathered comprehensive demographic data on all participants, encompassing age, sex, education level, marital status, and living arrangements. We also meticulously assessed several factors associated with COPD, including obesity^33^, alcohol consumption^34^, blood pressure^35^, blood sugar^36^, blood lipids^37^, asthma^38^, chronic kidney disease^39^, coronary heart disease^40^, arrhythmia^40^, depression^41^, gout^42^, and peptic ulcer^43^. These variables served as confounding factors and were duly adjusted for in our analysis.

The primary endpoint was the development of COPD, defined as forced expiratory volume in 1 s (FEV1)/forced vital capacity (FVC) < 0.7. Spirometry measurements of FEV1 and FVC were obtained using a MicroLab spirometer and Spida 5 software (Micro Medical Ltd., Rochester, Kent, UK), and administered by trained technicians following the 2005 technical standards of the American Thoracic Society and the European Respiratory Society^44^. Each participant underwent three lung function tests, all meeting the quality criteria standards (i.e., with differences within 5% or 100 mL), and the best result of the three tests was used for analysis. FVC-predicted (or FVC%-predicted) and FEV1-predicted (or FEV1%-predicted) values were calculated by dividing the measured values by the reference values based on formulas derived from the general Asian population regarding sex, age, and height. These population-specific formulas were integrated into the spirometry software, and individual participant details were entered to yield percent-predicted values. Post-bronchodilator measurements were not conducted.

### Statistical analysis

We conducted a longitudinal study, administering a follow-up survey to all subjects every 2 years, starting from their enrollment. The subjects were categorized into two groups: the exposure and no exposure to SHS groups. The categorical variables are represented as numbers and percentages, while continuous variables are presented as means along with standard deviations. Group comparisons were carried out using chi-square tests (for categorical variables) and independent t tests (for continuous variables). Subsequently, we employed Cox proportional hazards regression models (both univariate and multivariate) to explore the association between SHS exposure and COPD. The multivariate Cox proportional hazards regression models included an age-adjusted model, adjusted model 1, and adjusted model 2. Age adjustment was crucial due to its significance as a risk factor for COPD development in several studies^3,7^. This adjustment helped us better account for the influence of age on COPD risk, ensure comparability across age groups, and obtain more interpretable and accurate estimates of the association between SHS exposure and COPD development within our study population. Adjusted model 1 included variables that were statistically significant in the univariate analysis, while adjusted model 2 accounted for variables with clinical relevance and the potential to confound the association of COPD^33–43^. Differences were presented as relative risk (RR) and 95% confidence interval (CI). Additionally, we conducted a dose analysis to assess the impact of varying durations of SHS exposure. P value < 0.05 was considered indicative of statistical significance. The statistical analysis was performed using R (version 3.6.2, R Foundation for Statistical Computing, Wien, Austria) and SPSS (version 20.0, IBM Corp, Armonk, NY, USA).

## Results

### Baseline characteristics

A total of 1,453 men and 5,066 women were included in the study, and their baseline profiles are summarized in Table 1. There were 6,004 individuals in the no exposure group and 515 in the exposure group. Among the 515 participants exposed to SHS, the median weekly exposure was 1.2 h (interquartile range 0.5 to 5 h). SHS exposure was greater at home (n = 235, 46%) than at work (n = 94, 18%), while the remaining individuals were exposed in both settings (n = 174, 34%).

**Table 1:** Clinical characteristics of the study participants (n = 6519).

Characteristics	Secondhand Smoke Exposure		
	No Exposure, n = 6004	Exposure, n = 515	P value
Secondhand smoke exposure			
Location, %			
At home only	N/A	235 (46)	
At work only	N/A	94 (18)	
At both home and work	N/A	174 (34)	
Missing value	N/A	12 (2)	
Exposure hours per week, median (IQR)	N/A	1.2 (0.5 to 5)	
Demographic data			
Age, yr	51 ± 10	48 ± 10	< 0.001***
Women	4682 (78)	384 (75)	0.077
Body mass index, kg/m^2^	23.8 ± 3.5	24.2 ± 3.6	0.005**
Alcohol status, ever	169 (3)	34 (7)	< 0.001***
Physical activity, yes	2834 (47)	192 (37)	< 0.001***
Married, yes	5543 (92)	478 (93)	0.795
Educational status, ≧ college	3108 (52)	203 (39)	< 0.001***
Systolic blood pressure, mm Hg	117 ± 18	116 ± 18	0.242
Diastolic blood pressure, mm Hg	71 ± 11	71 ± 11	0.900
Comorbidities			
Hypertension	677 (11)	63 (12)	0.515
Diabetes mellitus	288 (5)	20 (4)	0.388
Dyslipidemia	443 (7)	41 (8)	0.600
Asthma	234 (4)	29 (6)	0.061
Chronic kidney disease	66 (1)	7 (1)	0.515
Coronary artery disease	68 (1)	4 (1)	0.659
Arrythmia	290 (5)	25 (5)	1.000
Peptic ulcer	922 (15)	79 (15)	1.000
Depression	203 (3)	14 (3)	0.521
Gout	140 (2)	11 (2)	0.879

IQR = Interquartile range.

** means P value < 0.01; and *** means P value < 0.001.

The SHS exposure group were younger, had a higher body mass index (BMI), and demonstrated a tendency toward a higher prevalence of alcohol consumption, along with a lower prevalence of physical activity and educational attainment compared to the no exposure group. No significant differences were observed in terms of sex, marital status, or the prevalence of comorbidities listed in Table 1 between the two groups.

### Associations between variables and the development of COPD

In the univariate analysis, BMI, systolic blood pressure (SBP), and diastolic blood pressure (DBP) demonstrated negative associations with the development of COPD (Table 2). Moreover, the risk of developing incident COPD was significantly higher the SHS exposure group than in the no exposure group (RR = 1.52; 95% CI 1.07 to 2.18; P value = 0.021). Conversely, no significant associations were identified between incident COPD and factors such as age, sex, or the comorbidities listed in Table 2.

**Table 2:** Parameters associated with incident COPD (n = 6519).

Parameters	Relative risk (95% CI)	P value
Age (per 1 year)	1.00 (0.98 to 1.01)	0.455
Women (*vs.* men)	0.95 (0.73 to 1.25)	0.729
Body mass index (per 1 kg/m^2^)	0.96 (0.92 to 0.99)	0.014*
Alcohol status, ever (*vs.* never)	0.76 (0.36 to1.61)	0.471
Physical activity, yes (*vs.* no)	0.95 (0.76 to1.20)	0.685
Married, yes (*vs.* no)	1.02 (0.66 to 1.58)	0.920
Living alone, yes (*vs.* no)	0.97 (0.60 to 1.56)	0.906
Education status (per higher level)	0.93 (0.84 to 1.05)	0.239
Systolic blood pressure (per 1 mmHg)	0.99 (0.98 to 0.99)	< 0.001***
Diastolic blood pressure (per 1 mmHg)	0.98 (0.97 to 0.99)	< 0.001***
Hypertension, yes (*vs.* no)	0.89 (0.61 to 1.29)	0.535
Diabetes mellitus, yes (*vs.* no)	0.78 (0.43 to 1.43)	0.428
Dyslipidemia, yes (*vs.* no)	0.68(0.38 to 1.21)	0.193
Asthma, yes (*vs.* no)	1.46 (0.89 to 2.38)	0.130
Chronic kidney disease, yes (*vs.* no)	0.91 (0.29 to 2.84)	0.871
Coronary artery disease, yes (*vs.* no)	0.92 (0.30 to 2.88)	0.890
Arrythmia, yes (*vs.* no)	0.62 (0.32 to 1.21)	0.161
Peptic ulcer, yes (*vs.* no)	0.87 (0.62 to 1.21)	0.406
Depression, yes (*vs.* no)	1.13 (0.62 to 2.06)	0.693
Gout, yes (*vs.* no)	1.33 (0.69 to 2.59)	0.397
Secondhand Smoke Exposure, yes (*vs.* no)	1.52 (1.07 to 2.18)	0.021*

COPD = Chronic obstructive pulmonary disease; CI = Confidence interval.

* means P value < 0.05; and *** means P value < 0.001.

### Association between SHS and the development of COPD

After a follow-up period of 48 months, COPD developed in 260 (4%) participants in the no exposure group and 34 (7%) participants in the exposure group (Table 3). After adjusting for confounding variables, the RR of incident COPD development was significantly higher in the exposure group than in the no exposure group (RR = 1.49; 95% CI 1.04 to 2.14; P value = 0.031) (Table 3).

**Table 3:** Relative risk for incident COPD by exposure of secondhand smoke after adjusting for confounders (n = 6519).

	No Exposure	Secondhand smoke exposure	P value
Presence of COPD, n (%)	260 (4)	34 (7)	
Age-adjusted relative risk (95% CI)	1.00 (reference)	1.51 (1.05 to 2.16)	0.025*
Adjusted model 1, relative risk (95% CI)	1.00 (reference)	1.55 (1.08 to 2.21)	0.017*
Adjusted model 2, relative risk (95% CI)	1.00 (reference)	1.49 (1.04 to 2.14)	0.031*

COPD = Chronic obstructive pulmonary disease; CI = Confidence interval.

Model 1 adjusts for age, body mass index, systolic blood pressure and diastolic blood pressure.

Model 2 adjusts for age, sex, body mass index, alcohol status, physical activity, marital status, living alone, educational status, systolic blood pressure, diastolic blood pressure, history of hypertension, dyslipidemia, diabetes mellitus, asthma, chronic kidney disease, coronary artery disease, arrythmia, peptic ulcer, depression, and gout.

* means P value < 0.05.

### Association between SHS Frequency and the Development of COPD

To further investigate the potential dose–response relationship between the duration of exposure to SHS and the risk of incident COPD, we analyzed the association using data from Table 4. The results revealed that each additional hour of weekly exposure to SHS was associated with a 1.03-fold increased likelihood of developing COPD (unadjusted RR = 1.03; 95% CI 1.01 to 1.05; P value = 0.013). This association remained significant after adjusting for various covariates, including age, sex, BMI, alcohol consumption, physical activity, marital status, living arrangements, educational level, SBP, DBP, and a range of medical conditions (adjusted model 2: RR = 1.03; 95% CI 1.00 to 1.05; P value = 0.027). Thus, these findings suggest that even relatively low levels of SHS exposure may contribute to an elevated risk of COPD development (Table 4).

**Table 4:** Relative risk for incident COPD according to weakly hours of secondhand smoke exposure.

Weekly hours of secondhand smoke exposure	Unadjusted relative risk (95% CI)	Age-adjusted relative risk (95% CI)	Adjusted model 1, relative risk (95% CI)	Adjusted model 2, relative risk (95% CI)
Per 1 h /week	1.03* (1.01 to 1.05)	1.03* (1.01 to 1.05)	1.03* (1.00 to 1.05)	1.03* (1.00 to 1.05)
P value	0.013	0.016	0.021	0.027

COPD = Chronic obstructive pulmonary disease; CI = Confidence interval.

Model 1 adjusts for age, body mass index, systolic blood pressure and diastolic blood pressure.

Model 2 adjusts for age, sex, body mass index, alcohol status, physical activity, marital status, living alone, educational status, systolic blood pressure, diastolic blood pressure, history of hypertension, dyslipidemia, diabetes mellitus, asthma, chronic kidney disease, coronary artery disease, arrythmia, peptic ulcer, depression, and gout.

* means P value < 0.05.

## Discussion

In this longitudinal study of Taiwanese never-smokers, a notable association was established between SHS exposure and an increased risk of COPD after accounting for confounding variables. In addition, there is a dose–response relationship between the duration of exposure to SHS and the risk of incident COPD, which demonstrates that an additional hour of exposure to SHS per week was associated with a 1.03-fold increased likelihood of developing COPD after adjusting for confounders. These findings demonstrate the interplay between SHS exposure and the incidence of COPD in the Asia–Pacific region.

SHS poses a significant public health threat due to its widely recognized detrimental effects on health, and it is important to note that no level of SHS exposure is risk-free^8–10,45,46^. Even brief encounters with SHS can have considerable adverse impacts on the respiratory, cardiovascular, immune, and endocrine systems^45,46^. Numerous studies have explored the connection between COPD and SHS, of which many have primarily focused on the effects of SHS exposure in patients already diagnosed with COPD^18,47,48^. These investigations have reported associations between exposure to SHS with unfavorable patient-centered outcomes, such as dyspnea and chronic cough. In addition, SHS exposure has been linked to a reduced disease-specific quality of life in COPD patients who are not current smokers^18,47^. Furthermore, SHS has been shown to have detrimental effects on active smokers who have already been diagnosed with COPD^48^.

In line with our findings, numerous investigations have consistently emphasized a notable increase in the risk of COPD linked to SHS exposure, with odds ratios (ORs) ranging from 1.31 to 2.24^20,49,50^. An analysis conducted by Yin and colleagues of 6,497 Chinese individuals who had never smoked revealed that exposure to 40 h of passive smoking per week for more than 5 years was correlated with a 48% higher odds of spirometrically defined COPD^20^. Similarly, a cross-sectional study conducted in the United States involving 2,113 adults reported a 36% greater odds of developing COPD associated with exposure to SHS^49^. Three systematic reviews and meta-analyses, incorporating data from 21, 15, and 8 studies respectively, provided evidence supporting a correlation between SHS exposure and the risk of COPD, with relative risks of 1.44 (95% CI 0.67 to 3.12), 2.02 (95% CI 1.52 to 2.67), and 1.66 (95% CI 1.38 to 2.00), respectively^14,51,52^.

In contrast, other studies have reported a different perspective, with no direct link between exposure to SHS and the risk of developing COPD^19,53–55^. For example, a 20-year longitudinal study of 3,011 European adults—both never-smokers and ex-smokers—suggested a potential association between SHS exposure and respiratory symptoms; however, it did not establish a definitive link to changes in lung function or the occurrence of COPD^55^. Similarly, a study conducted by Kim et al. in Korea found no noticeable difference in the prevalence of COPD between never-smokers exposed and not exposed to SHS^53^. Moreover, a systematic review and meta-analysis of observations from seven prospective cohorts found no statistically significant evidence of an association between SHS exposure and COPD (RR = 1.21; 95% CI 0.93 to 1.57)^14^.

The disparities in outcomes noted among studies may originate from variances in study populations and inclusion criteria, notably accounting for the incorporation of ex-smokers in certain studies, potentially altering susceptibility to the effects of SHS. In addition, covariates integrated into the analyses may exacerbate potential bias and exert influence on the observed results. Furthermore, the prevalent utilization of cross-sectional designs in preceding studies necessitates careful consideration when drawing conclusive inferences. Therefore, the findings of this community-based, longitudinal investigation elucidating the association between SHS and COPD harbor significant potential for informing and shaping future research endeavors in this domain.

We also found a dose–response relationship between SHS and COPD, with a 1.03-fold increased risk of developing COPD with each additional hour of SHS exposure per week. Similar to our findings, several previous studies have also suggested a correlation between a longer duration of exposure to SHS with a higher prevalence of COPD^50,56^. In study conducted in Estonia, individuals exposed to more than 5 h of SHS per day (equivalent to over 35 h per week) exhibited a 1.54-fold higher risk (95% CI 1.13 to 3.00) of physician-diagnosed chronic bronchitis or emphysema^56^. Conversely, for those exposed to 1 to 5 h per day (equivalent to 7 to 35 h per week), the risk was lower (OR = 1.16; 95% CI 0.88 to 1.53)^56^. Similarly, a study from England reported that greater exposure to passive smoke was independently associated with an elevated risk of COPD, with an adjusted OR, 1.05 (95% CI 0.93 to 1.18) for 1 to 19 h of exposure per week, and 1.18 (95% CI 1.01 to 1.39) for 20 or more hours of exposure per week^50^.

SHS consists of 15% mainstream smoke, inhaled and exhaled by smokers, and 85% sidestream smoke emitted from the burning tip of a cigarette. Sidestream smoke is notably more toxic than mainstream smoke, and both contain a complex array of over 4,000 chemical compounds, including carcinogens and respiratory toxins^57^. Woodruff et al.^58^, and more recently, Goldklang et al.^59^, investigated into SHS-induced emphysema mechanisms using mouse models. Their research demonstrated that SHS exposure triggers an increase in alveolar macrophage recruitment and activation markers, mirroring responses observed in human smokers and ultimately leading to emphysema^58^. SHS exposure has also been demonstrated to contribute to the degradation of elastin and lung structure, further bolstering its association with the development of emphysema^57^. Recent research has also linked SHS to thicker airway walls in COPD patients, correlating with respiratory symptoms^47,48^. Taken together, these findings may explain the mechanisms underlying the association between chronic SHS exposure and COPD.

Some studies have investigated the short-term impact of SHS exposure on lung function and COPD, and others have examined the long-term effects. Notably, the TackSHS study found that acute SHS exposure (within 60 min) had no significant impact on COPD symptoms or spirometric indices after 24 hours^60^. In addition, Flouris et al. observed that decreases in lung function caused by 1 h of exposure to SHS receded within 60 min. However, they also found that elevated inflammatory cytokine levels persisted for at least 3 h post-SHS exposure, suggesting chronic low-grade inflammation in individuals consistently exposed to SHS^45^. This underscores that even brief SHS exposure can initiate mechanisms leading to COPD development.

Anti-smoking policies implemented worldwide have successfully reduced SHS exposure and improved respiratory symptoms, especially among bar and other hospitality workers^19,61,62^. A systematic review conducted by Callinan et al. found that the introduction of a smoking ban led to improved health outcomes through a reduction in SHS exposure, particularly in relation to cardiovascular disease^61^. However, consistent evidence on the reduction in active smoking is lacking, and exposure to SHS at home and in private cars has not changed significantly since implementing smoking bans^63^. These findings emphasize the numerous harms of SHS, and underscore the health-promoting effects of smoking bans. Consequently, our results and those of the previous studies reiterate the importance of smoking cessation and provide a foundation for governments to develop pertinent anti-smoking policies.

This study has several limitations. First, we assessed SHS exposure through self-reported surveys, introducing the potential for recall bias. To more comprehensively evaluate the dose-related association, obtaining information on the number of cigarettes smoked by others or assessing biomarkers such as urine cotinine or NNAL level could be valuable. It is important to note that these biomarkers are suitable for assessing short-term and medium-term exposure, respectively^47^. Second, we were unable to gather information on changes in exposure during the follow-up period and the total years of SHS exposure due to the simplicity of the survey questions. Given that COPD is associated with continuous and long-term exposure to risk factors, tracking changes in exposure and total exposure duration is crucial. A study with an extended follow-up period could provide further insights. Third, the participants may have had additional risk factors contributing to COPD that were not adjusted for as confounders in our study, such as occupational exposure or residing in industrial areas. Considering the potential impact of urban air pollution on incident COPD, adjustment for urbanization level may also be necessary. Lastly, no post-bronchodilator measurements were conducted; however, we did account for self-reported asthma in the adjustments.

In conclusion, the findings of this longitudinal study with 4 years of follow-up suggest a potential causal effect between SHS and COPD among non-smokers. We also identified a dose–response relationship between SHS exposure and the development of COPD. While randomized controlled trials remain the gold standard for establishing causality, they may present ethical challenges. Therefore, future prospective multi-racial, large-scale studies should be undertaken to further validate the impact of SHS on the development of COPD in individuals who have never smoked.

## Data Availability

The data underlying this study are from the Taiwan Biobank. Due to restrictions placed on the data by the Personal Information Protection Act of Taiwan, the minimal data set cannot be made publicly available. Data may be available upon request to interested researchers. Please send data requests to Szu-Chia Chen, Division of Nephrology, Department of Internal Medicine, Kaohsiung Medical University Hospital, Kaohsiung Medical University.
